# Releases of Asian houbara must respect genetic and geographic origin to preserve inherited migration behaviour: evidence from a translocation experiment

**DOI:** 10.1098/rsos.200250

**Published:** 2020-03-18

**Authors:** Robert J. Burnside, Claire Buchan, Daniel Salliss, Nigel J. Collar, Paul M. Dolman

**Affiliations:** 1 School of Environmental Sciences, University of East Anglia, Norwich Research Park, Norwich, Norfolk NR4 7TJ, UK; 2 BirdLife International, Pembroke Street, Cambridge CB2 3QZ, UK

**Keywords:** bustard, migratory orientation, migratory strategy, population reinforcement, population reintroduction

## Abstract

Maintaining appropriate migratory strategies is important in conservation; however, translocations of migratory animals may alter locally evolved migration behaviours of recipient populations if these are different and heritable. We used satellite telemetry and experimental translocation to quantify differences and assess heritability in migration behaviours between three migratory Asian houbara (*Chlamydotis macqueenii*) breeding populations (640 km range across eastern, central and western Uzbekistan). Adults from the eastern population migrated twice as far (mean = 1184 km ± 44 s.e.) as the western population (656 km ± 183 s.e.) and showed significantly less variation in migration distance than the central population (1030 km ± 127 s.e.). The western and central populations wintered significantly further north (mean: +8.32° N ± 1.70 s.e. and +4.19° N ± 1.16 s.e., respectively) and the central population further west (−3.47° E ± 1.46 s.e.) than individuals from the eastern population. These differences could arise from a differing innate drive, or through learnt facultative responses to topography, filtered by survival. Translocated birds from the eastern population (wild-laid and captive-reared, *n* = 5) migrated further than adults from either western or central recipient populations, particularly in their second migration year. Translocated birds continued migrating south past suitable wintering grounds used by the recipient populations despite having to negotiate mountain obstacles. Together, this suggests a considerable conserved heritable migratory component with local adaptation at a fine geographic scale. Surviving translocated individuals returned to their release site, suggesting that continued translocations would lead to introgression of the heritable component and risk altering recipient migration patterns. Conservation biologists considering translocation interventions for migratory populations should evaluate potential genetic components of migratory behaviour.

## 1. Introduction

Maintaining successful migratory strategies in the face of environmental change is a fundamental challenge facing conservation biology [1,2]. Conservation translocations must ensure founders show appropriate physiological, behavioural and genetic characteristics [3]; increasing use of translocations to reintroduce or reinforce migratory populations highlights the need to understand how translocated individuals establish their migratory behaviour [4]. In some vertebrates, migration behaviours are culturally transmitted, allowing translocated individuals to learn traditional routes from conspecifics [5,6] or humans [7]. But in many species, particularly where first migration occurs in the absence of experienced individuals, migratory traits are innate [4,8] with a strong heritable component [9–11]. For such species, translocation for reinforcement using individuals from an allopatric source population may disrupt the recipient population's migration strategy, potentially altering fitness [12–14], while translocation for reintroduction of extirpated populations may not replicate historic migration strategies [15]. Even where phylogeographic analyses suggest large-scale population genetic homogeneity this could mask finer-scale adaptation of behaviours under strong local selection. When developing a translocation programme for migratory species, an experimental investigation is, therefore, required to assess whether the interplay of facultative and heritable behaviour leads translocated individuals to establish appropriate migration routes.

Migratory species from all vertebrate groups are threatened [16,17], with migrant birds more at risk than residents [18,19], through phenological mismatches [20] and cumulative anthropogenic threats across breeding and wintering ranges and along migratory routes [21]. Diverse endangered migratory species are already subject to *ex situ* management and release [6,7,22], but success following release depends on the migratory pathways adopted [15,23]. Novel migrations may facilitate responses to environmental change [24,25]; particularly when current routes are constrained, their disruption may assist adaptation to potentially suitable landscapes and climates lying beyond current pathways. However, if newly expressed migration routes achieve lower survival [26] or productivity (e.g. through carry-over effects [27]) relative to established strategies, this may reduce population viability [28] and potentially result in catastrophic population losses [29]. Conservation and welfare considerations combine to require that experimental evidence in an adaptive management approach is sought to avoid the potential negative consequences of translocations.

To explore the importance of genetic origin for reinforcement initiatives involving migratory species, we used the migratory Asian houbara (*Chlamydotis macqueenii*) as a model system, examining populations along a longitudinal gradient within the Central Asian deserts. The species undertakes long-distance migrations and is subject to large-scale captive breeding and release programmes to reinforce threatened migratory populations throughout its range [30]. Observational studies suggest that migration orientation and distance are heritable [31,32] and differ across the range [31], with juveniles migrating independently of their mothers [32] and probably also of experienced adults, as they leave earlier than adults, migrate more slowly flying fewer kilometres per day [32], and spend longer on stop-overs [33]. Treating migratory Central Asian populations as a single unit for management therefore risks homogenizing and potentially compromising population-specific migratory strategies [31].

To examine whether local migratory populations of Asian houbara retain distinct innate migration strategies, despite gene-flow and minimal population structure [34], we examined wild and translocated migration patterns in three breeding populations across 640 km (58.05°–63.90° E) of desert in Uzbekistan, a fine-grained scale relative to the full breeding span (4460 km) of migratory Central Asian populations from Iran/Kazakhstan to China (51°–106° E). We experimentally translocated ‘head-started’ individuals (captive-reared from wild-laid eggs) of eastern origin into central and western populations. Using satellite telemetry, we compared the migration routes of the source and recipient populations, and assessed the extent to which released birds replicated the migration patterns of recipient wild populations.

## 2. Methods

### 2.1. Study system

**Figure 1.**
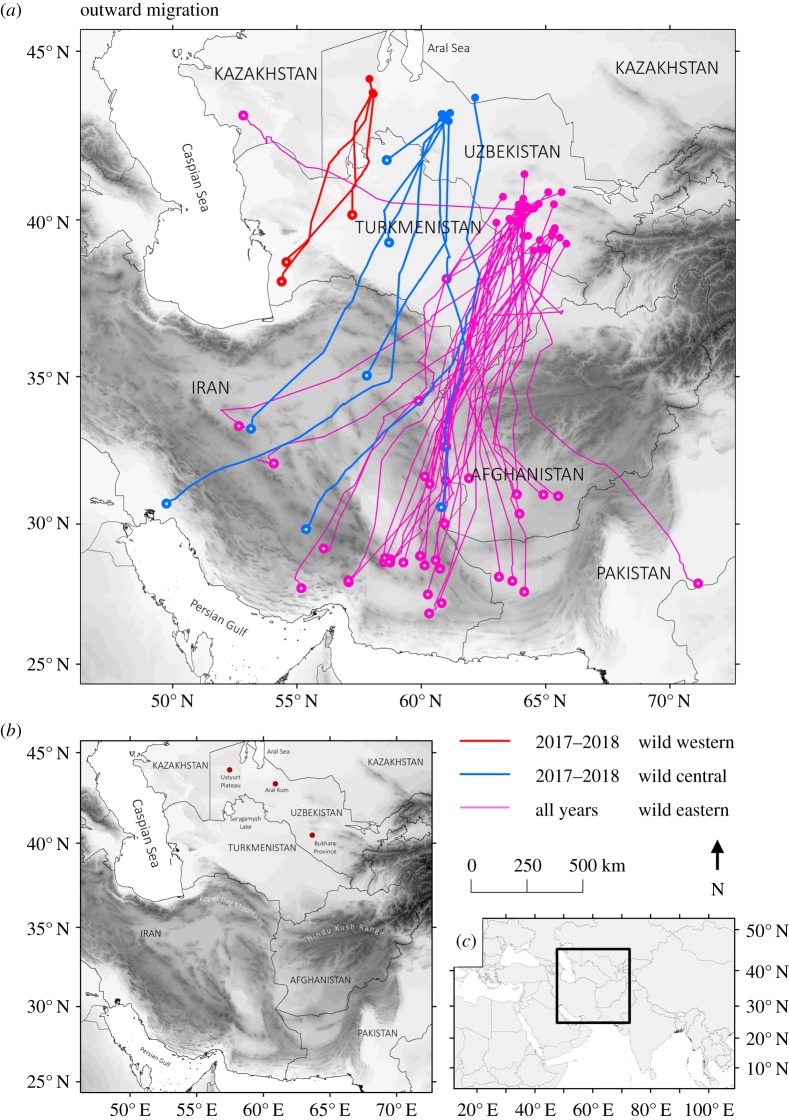
Migration routes (*a*) used by wild Asian houbara adults from three different breeding populations in Uzbekistan (Bukhara: eastern, Aral Kum: central, Ustyurt: western). Starting points of tracks are indicated by solid dots, while the end of tracks are shown as larger dots with a white central dot; only one migration route is shown for each individual for clarity. (*b*) Topographic detail including obstacles (water bodies and mountains with darker shades representing higher elevations) to migration paths in Central Asia. (*c*) The map in global position (*a*).

Within Uzbekistan we examined a west–east longitudinal range: from the ‘western’ population in the Ustyurt Plateau (43.87° N, 58.05° E) located towards the species' southwestern range limit; a ‘central’ population separated by the Aral Sea basin (figure 1) and lying approximately 200 km east in the Aral Kum (42.88° N, 61.31° E); and an ‘eastern’ population in the Kyzylkum desert (40.40° N, 63.90° E), 640 km east of the Ustyurt and 450 km east of the Aral Kum. We considered these populations functionally disjunct: a satellite telemetry study of 170 adult years (from 74 individuals) from the eastern population showed only one instance of short-term dispersal to the central population (an adult female from Bukhara moved west and re-nested but subsequently returned to the eastern population), while no individuals moved to the western population. No individual moved from the central or western to the eastern population in 22 adult years (12 individuals) of satellite telemetry. Wild adults and juveniles from the eastern population primarily migrate along a south/southwesterly route [32] passing the Iran/Turkmenistan border between the Hindu Kush (maximum 7690 m elevation) and the Kopet Dag mountains within the Turkmen-Khorasan range (maximum 3190 m: forming a broad west–east topographic obstacle to the north–south migration), to winter in southern Iran and Pakistan (figure 1). Migration routes of central and western populations had not been characterized previously, but we *a priori* hypothesized that western individuals may follow a similar migration route to Asian houbara breeding to the northwest (on the Turanian Plain in western Kazakhstan), which initially migrate south over the Ustyurt Plateau, then southwest to west crossing the Kopet Dag mountains, which do not appear to act as a barrier to their migration [31,33], to winter southwest of the Caspian Sea predominantly in southern Iran and Iraq.

### 2.2. Migratory data acquisition

Wild breeding adults were captured during the breeding season and fitted with back-mounted solar-powered satellite transmitters (PTTs) programmed to record 5 (30 g PTT) or 12 (45 g PTT) GPS locations per day (accuracy within 18 m; further details in electronic supplementary material, methods S1). Transmitters weighing less than 3% of bird body mass are considered to have no detrimental effect on houbara [31] and do not affect adult female breeding probability, clutch size, egg size or nest success [35]. All tracked wild adults had previously completed at least one return migration.

For head-starting and translocation, 11 wild clutches were collected from the eastern population (all from within a 30 km radius) in spring 2017, artificially incubated and hand-reared in captivity (electronic supplementary material, methods S1). Twelve individuals (eight males and four females sexed on size dimorphism) were randomly assigned to the central and western populations (six each): three mothers each contributed two chicks, of which one sibling was allocated to each treatment; the remaining individuals were each from different mothers. Translocated birds were released at six months old in mid-September (as earlier release during hot August weather reduces survival [26]) and would have approximately one month of free-living to develop physiology, flight power and endurance before migration onset, if this occurred at the same time as their eastern (source) population (mean = 21 October [32]).

Migration strategy can be age-related in birds [36] and a comparison of translocated head-started birds with wild juveniles in the recipient populations would be the ideal. In this study, we compared translocated juveniles to adults of unknown age, as it was not possible to trap wild juveniles in the two recipient areas. However, previous studies found no difference in migration distance or initial bearing, for larger samples of wild juvenile and adult Asian houbara [32,33], while juveniles that survived their first winter returned to their first wintering sites in subsequent migrations [31,32], consistent with the lack of population-scale age-dependent migration. Therefore, we consider it appropriate to compare migration of translocated juveniles to adults of the recipient population. However, one potential limitation in our experiment is that captive-rearing may affect first-winter migratory behaviour, as head-started juveniles previously migrated shorter distances than wild juveniles [32], probably owing to physiological limitation and the physical demands of flapping flight. We acknowledge this may curtail the migratory distance of captive-reared translocated juveniles relative to that of wild adults.

### 2.3. Analysis

**Figure 2.**
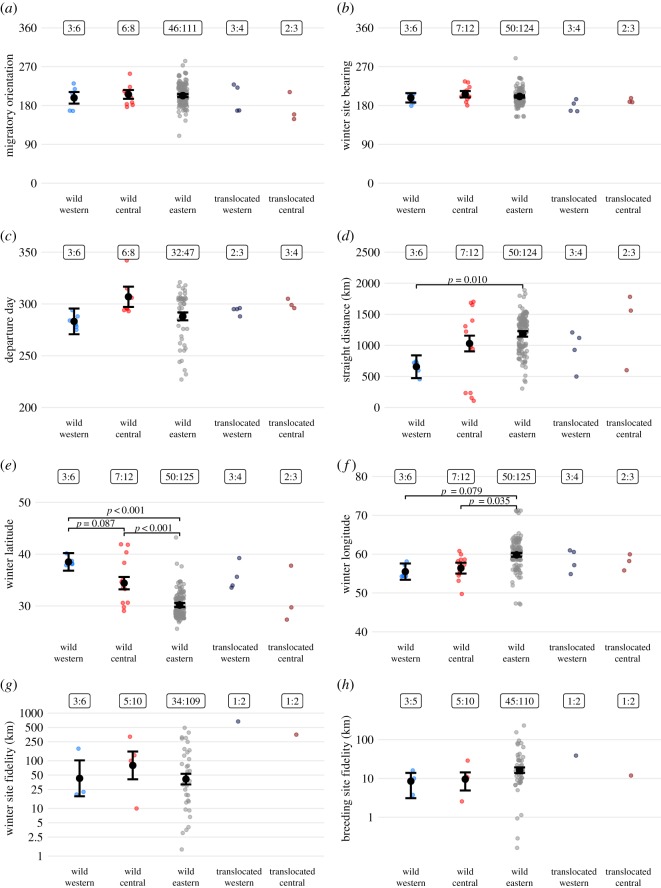
Migration metrics for three wild populations (eastern, central and western) of Asian houbara in Uzbekistan and two groups of head-started birds originating from the eastern population and translocated to the western and central populations. Background dots show the data, while the black points are model estimated means with standard error bars; tests between wild groups are indicated by horizontal bars and *p*-values. Translocated groups were not statistically tested. Boxes give the number of individuals and the total number of migration tracks for each group.

We focused analyses on migration traits considered likely to be under genetic control (and hence conserved): initial orientation during the first migration step (hereafter ‘first-step bearing’); departure date from the post-breeding grounds; bearing from post-breeding area to wintering-site; wintering-site latitude and longitude; migratory distance (the straight-line displacement from post-breeding to wintering-site); wintering-site fidelity; and fidelity after return migration to breeding/release site [31,32,37]. We did not compare stop-over locations and timings, speed or migratory efficiency, as these are probably influenced by weather and certainly by age [32,33]. Only tracks that could provide reliable estimates of each migratory parameter were included (sample sizes are shown in figure 2; details of telemetry data processing and criteria for identifying stop-over and wintering sites are described in electronic supplementary material, methods S1).

Migration metrics were compared between the three populations using generalized linear mixed models (GLMMs), incorporating individual identity as a random effect. We considered the effect of population identity to be supported if incorporating that term reduced the Akaike's information criterion adjusted for small sample size (AICc) value by ≥2 units relative to the null model [38], and subsequently tested pairwise differences between populations, controlling for experiment-wide error using a Tukey honestly significant difference (HSD) test. All GLMMs had a normal error structure, as bearings ranged between 120° and 280°; measures of site fidelity (breeding and wintering) were log-transformed. Model residuals were checked for normality and homoscedasticity.

The variance of each migration metric was compared between populations using pairwise *F*-tests (after averaging each metric per individual to avoid pseudo-replication), controlling for experiment-wide error by a Bonferroni procedure. In all tests, *p* < 0.05 was interpreted as a significant difference, and *p* < 0.1 as marginally different (noting the small sample sizes of some wild groups). Translocated groups were qualitatively compared to data for wild groups owing to small sample sizes.

Analyses considered both males and females from the eastern population, but only males from the central and western populations as it was not possible to trap females in these areas. Previous analysis found no difference between male and female adult houbara for first-step bearing, departure date, migration distance, wintering latitude, or breeding-site fidelity [32,33]. However, as a precaution we also examined analyses repeated while excluding females from the eastern population.

## 3. Results

Model selection tables and *F*-test results can be found in electronic supplementary material, appendix S2.

### 3.1. Characterizing wild strategies

Adult migration strategies differed between the three populations (figures 1 and 2; electronic supplementary material, figure S1), with western individuals wintering in Turkmenistan, central individuals wintering from Turkmenistan to southern Iran, and eastern individuals wintering mainly across Afghanistan, Iran and Pakistan and infrequently in Iraq and Turkmenistan. All populations had a similar migratory orientation (figure 2*a*,*b*), in terms of both first-step bearing (means ± s.e.: western 197.96° ± 13.34, central 205.73° ± 10.25, eastern 203.61° ± 3.33; ΔAICc = −3.86 on removal of the population term) and wintering-site bearing (mean ± s.e.: western 197.96° ± 10.95, central 201.62° ± 2.61, eastern 206.22° ± 7.46; ΔAICc = −3.74). Variance in first-step and wintering-site bearings was also similar between populations (all *F*-tests, *p* = 1; electronic supplementary material, appendix S2). Departure day from the post-breeding areas did not differ between populations (mean ± s.e.: western 20 October ± 12, central 4 November ± 10, eastern 15 October ± 4; ΔAICc = −19.31 on removal of the population term; figure 2*c*) and variance in departure date was similar across populations (all *F*-tests, *p* > 0.44; electronic supplementary material, appendix S2). However, populations differed in wintering latitude (ΔAICc = 22.57 on removal of the population term), with individuals from the eastern population wintering further south (mean latitude = 30.17° ± 0.41 s.e.) than those from the central (34.36° ± 1.16 s.e., *z* = 3.61, *p* < 0.001) and western (38.49° ± 1.70, *z* = 4.90, *p* < 0.001) populations, and western individuals marginally (*z* = 2.09, *p* = 0.087) further north of those from the central population (figures 1 and 2*e*). Overall migration distance also differed between populations (figure 2*d*; ΔAICc = 4.31 on removal of the population term). Adults from the eastern population migrated nearly twice as far (mean = 1184 km ± 44 s.e.; figures 1 and 2*d*) as those from the western population (656 km ± 183 s.e., *z* = 2.88, *p* = 0.001) but had similar variance (*F*_49,2_ = 0.350, *p* = 1). Adults from the central population migrated an intermediate distance (1030 km ± 127 s.e.) that did not differ significantly from either the western (*z* = −1.75, *p* = 0.18) or eastern (*z* = 1.22, *p* = 0.43) populations but with significantly greater variance than eastern individuals (*F*_49,6_ = 0.16, *p* < 0.001; electronic supplementary material, appendix S2). Populations also differed in wintering longitude (figure 2*f*; ΔAICc = 4.77 on removal of the population term), with individuals from the central population (mean longitude = 56.40° ± 1.42 s.e.) wintering significantly further west (*z* = −2.453, *p* = 0.035) and the small sample of western birds (55.45° ± 2.08 s.e.) marginally further west (*z* = −2.130, *p* = 0.079) than those from the eastern population (59.87° ± 0.50 s.e.). The variance in wintering latitude (figure 2*e*) of individuals from the central population was marginally greater than those from the eastern population (*F*_49,6_ = 0.341, *p* = 0.096), but other pairwise comparisons of wintering latitude variance did not differ (both *F*-tests, *p* > 0.169) and the variance in wintering longitudes was similar across the populations (all *F-*tests, *p* = 1; electronic supplementary material, appendix S2). One male from the larger sample of eastern individuals undertook an anomalous migration, moving northwest to winter in Kazakhstan on the Caspian Sea (figure 1).

Wintering-site fidelity, the mean distance between successive annual wintering sites, was similar across individuals from all three populations (overall mean = 44.7 km [28.6–70.0 95% CI]; ΔAICc = −3.87 on removal of the population term; figure 2*g*; electronic supplementary material, appendix S2 and figure S2) and was fine-grained compared to both the scale of habitat extent within wintering ranges and the distances travelled during outward migration (overall mean = 1258 km ± 401 s.d.). Within-population variance in wintering-site fidelity (among individuals) was also similar between populations (all *F*-tests, *p* = 1; electronic supplementary material, appendix S2). Breeding-site fidelity was similar across populations (overall mean = 10.2 km [8.9–13.0 95% CI]; ΔAICc = −2.25 on removal of the population term; figure 2*h*). Across all three populations, the 20 wild adult males returned to their display areas, but two of the 33 eastern females changed breeding site between years by greater than 200 km (figure 2*h*). The variance in breeding-site fidelity was similar across populations (all *F*-tests, *p* > 0.646; electronic supplementary material, appendix S2). Results were unchanged when the eastern sample was restricted to males.

Routes taken by the wild birds, with some exceptions, avoided crossing the Kopet Dag or the Hindu Kush mountains; for the eastern population, all except one track passed through the gap between these ranges, while individuals from the western population stopped before reaching the Kopet Dag rather than following the route of the Turanian Plain breeding population, which passes through or over this range. For the central population, four individuals flew over the Kopet Dag, one used the same gap as the eastern population, and two wintered in Turkmenistan to the north of the mountain range.

### 3.2. Behaviour of released translocated birds

**Figure 3.**
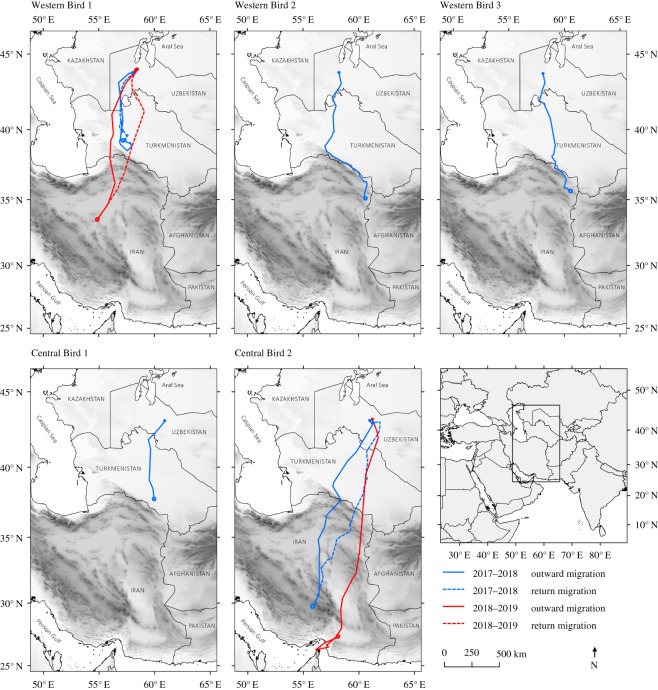
Outward (autumn) and return (spring) migrations of head-started (wild-laid, captive-reared) Asian houbara translocated from their eastern source population into central (two individuals) and western (three individuals) Uzbekistan. One translocated individual from each release returned and subsequently completed a second migration (Central Bird 2, Western Bird 1). Filled points indicate summer locations, hollow points indicate either wintering location (Western Bird 1, Central Birds 1 + 2) or the furthest stopping point reached on migration for individuals that died *en route* or after arrival (Western Bird 3 and Western Bird 2, respectively).

Of 12 head-started birds translocated from the eastern population, three released into the western population and two released into the central population survived to initiate migration in autumn 2017 (comprising three females, two males, all from different mothers). Of the five individuals that initiated migration, four survived to reach a wintering location (according to criteria of stop-over duration), and two (one from each recipient population) completed return migration in spring 2018 and migrated again the following winter (figure 3: Western Bird 1 and Central Bird 2). Departure dates and first-step bearings of the five translocated individuals were similar to those of all three wild breeding populations (figure 2) but subsequent movement steps during their first autumn migration appeared to be affected by the Kopet Dag mountains on the Turkmenistan–Iran border (figure 3).

Notably, all three eastern birds translocated into the western population that survived to migrate travelled approximately 330 km further south (mean difference = 2.9° N ± 2.6 s.e.) than wild adults from the recipient population (figure 2*d*,*e*). Their first-winter outward routes showed an apparent mix of conserved orientation and facultative response to the Kopet Dag. One individual encountered the mountains but did not cross and turned back north to remain and winter in Turkmenistan (figure 3: Western Bird 1), while two travelled southeast along the mountain range to its end at the Iran–Afghanistan border, and then persisted south (figure 3: Western Birds 2 + 3). Of these two, one survived 17 days after stopping migration movements and was considered to have reached its wintering-site (at a latitude of 33.98° N), while the second died (latitude 35.56° N) 6 days after stopping migratory movements and may, therefore, not have fully reached its wintering-site. Interestingly, the individual that encountered but did not cross the mountain range in its first winter initially followed a similar migration route in its second winter, but then flew over the Kopet Dag to Iran, thereby changing wintering site to a much lower latitude (710 km further south, first winter 39.24° N, second winter 33.53° N, figure 3: Western Bird 1; also see electronic supplementary material, figure S2).

Of the two eastern birds translocated into the central population that survived to migrate, one (Central Bird 1) wintered in Turkmenistan and survived to the following March. The second reached a site in Iran further south (29.72° N) than the central population mean (34.36° N), again showing an initial deflection by the mountains, but on its second autumn migration it followed a more direct route (similar to its previous return path) that avoided the Kopet Dag and brought it to the southern coast of Iran (27.35° N, further south than all central individuals), before it turned north and died.

Both surviving translocated birds (Central Bird 2 and Western Bird 1) showed fidelity to their release areas, initially returning to within 11.9 and 38.8 km, respectively (figure 3); after its second migration, Western Bird 1 (a female) subsequently survived a second migration, again returning to breed in the same location.

## 4. Discussion

Three wild breeding populations across a longitudinal gradient spanning only 640 km (14% of the 4460 km breeding range of migratory Asian houbara) had similar timing and orientation but differed in migratory distance and wintering latitude. Such differences in adult strategy could result from innate differences, learnt facultative responses to region-specific topography and environment along potential routes, or differential survival filtering out first year birds following sampling [39]. However, although based on limited sample size (*n* = 5), migratory behaviour of experimentally translocated birds indicated a potentially considerable heritable component to the differing migration strategies. This would signify finer-scale adaptation than suggested for Asian houbara by neutral genetic markers [34] or broader-scale migration studies [31,33].

Translocated birds showed a clear drive to continue migrating south similar to their source population and migrated further than individuals from their recipient populations, which is strongly indicative of an inherited and innate migration trait. The heritability of migration distance is also supported given that translocated birds continued to fly south despite (i) passing suitable wintering sites used by the recipient population, (ii) the time and energy required to negotiate the Kopet Dag mountain barriers, and (iii) the expected weaker physical condition of captive-bred juveniles relative to wild adults [32]. Furthermore, though quasi-anecdotal, one translocated juvenile that failed to cross the mountain range in its first winter subsequently migrated further south surmounting this mountain obstacle in its second winter, to winter further south than the recipient population. This is notable, as it runs counter to the explanation that experienced adult western birds winter north of the mountains because they have learnt this is favourable, and further emphasizes the potential importance of a heritable drive to continue south that differed between translocated individuals and the recipient population.

The most probable mechanisms for innate control of migration distance are the duration of migratory activity and/or an innate latitudinal cue to settle [32]. It is not possible to rule out a contribution from social cues, but this appears unlikely given the slowness of the migration of the translocated birds and evidence suggesting that they do not follow adults [31–33]. Ultimately—provided mountain ranges could be overcome or avoided—all southerly routes led to Iranian wintering grounds with suitable habitat and climate. Consequently, translocated individuals were able to survive the winter despite not following the migratory paths of their recipient population; but further research is needed to determine how survival rates and breeding productivity might differ between translocated birds and recipient populations. The migratory strategies of the central and eastern Uzbekistan populations have been maintained despite potential gene-flow (of subadult or late-returning males) from larger populations within the migratory flyway [31]. By contrast, western birds showed greater geographic isolation from this flyway and greater local adaptation in migration strategy. We predict that coarser-scale translocations across the migratory range and flyway, with individuals from Mongolia and China having an initial westerly trajectory before reaching Kazakhstan and turning south [31], would be even more disruptive than our fine-scale experiment. Releases into western Kazakhstan by the Sheikh Khalifa Houbara Breeding Centre situated in eastern Kazakhstan led to reports (R. Sheldon 2019, personal communication) during winter 2019–2020 of exhausted (and caught by hand) captive-bred Asian houbara occurring in Lankaran province of Azerbaijan (darvic ring R491) and the Talesh (darvic ring T390) and Gilan (darvic ring C741) provinces of Iran, all situated on the western coast of the Caspian Sea and highly anomalous for wild houbara migration. The most likely explanation is that those birds crossed the Caspian on a bearing consistent with an innate south/southwest migration path of birds from eastern Kazakhstan and unlike those of birds from the Turanian Plain, which would normally travel due south to avoid crossing the Caspian [31].

Translocated birds showed strong fidelity to release sites, allowing them to integrate with and reinforce the recipient population. Consequently, genotypes of translocated individuals are likely to introgress into recipient populations. Where released genotypes are even slightly less fit, supplementation can reduce population sizes and genetic diversity over the long term [28]. Large-scale releases of individuals of different geographical origins risk overwhelming locally adapted genotypes of recipient populations whatever the latter's fitness advantage [40,41].

Currently, there is no agreement in the Convention on Migratory Species or between range states on sustainable management and translocation strategies for this heavily hunted migratory species, with multiple stakeholders releasing birds throughout its range [42]. There are seven large-scale breeding centres distributed within Arabia and Central Asia (from at least five different organizations): those in Uzbekistan and Kazakhstan release captive-bred birds within their source populations [30,43], but other centres in the Middle East have released more widely, including into Jordan, UAE, Kingdom of Saudi Arabia, Kuwait, Bahrain, Qatar, Pakistan, Kazakhstan and Uzbekistan [42,43], involving thousands of translocated birds derived from breeding stock established from both resident (Afghanistan, Yemen and Iran) and migratory (Kazakhstan) Asian populations, with no information provided on the degree to which accessions are maintained as separate breeding lines, or the geographic match between released birds and recipient populations [44]. Our study suggests that conservation reinforcement should avoid interbreeding resident and migratory stocks or releasing birds outside their geographic origin, in order to preserve latent population structure potentially vital to the migratory capabilities of locally adapted populations. Releasing birds sourced from the same geographic origin is a feasible and precautionary approach, as many populations within Central Asia have the potential to be managed sustainably with a reduced need for captive-bred supplementation, provided hunting pressure is reduced [30].

Where migratory behaviour is known or suspected to be heritable, translocations should respect the fine-scale geographical structure of source and recipient populations. When this is not feasible (due, for example, to small population size), trial releases should be undertaken to assess both the potential differences between source and recipient populations and the effectiveness of further releases. The evidence presented here indicates the vital importance of experimental studies to evaluate the migratory behaviour of translocated individuals before scaling up interventions to a conservation reinforcement.

## Supplementary Material

Supplementary tables

Reviewer comments

## Supplementary Material

Supplementary methods

## Supplementary Material

Supplementary Figure

## Supplementary Material

Supplementary data

## Data accessibility

The datasets supporting this article have been uploaded as part of the electronic supplementary material.
