# Supplementary material for: Releases of Asian houbara must respect genetic and geographic origin to preserve inherited migration behaviour: evidence from a translocation experiment
Source: R Soc Open Sci. 2020 Mar 18;7(3):200250. doi: 10.1098/rsos.200250 (PMC7137974; doi:10.1098/rsos.200250)
Supplement: Supplementary tables [file rsos200250supp1.docx]

**Supplementary Material**

**Inherited behaviour of translocated animals risks altering migration patterns in recipient populations.**

**Appendix S1: Tables of sample sizes, model selection, model estimated group differences and variance tests.**

1. **Initial migratory orientation**

**Table S1.** Sample sizes for **initial migratory orientation** of satellite tracked wild Asian houbara from three populations within Uzbekistan showing the number of individuals monitored and the total number of complete migration tracks used in analysis.

|  | *Sample Sizes* | | |
| --- | --- | --- | --- |
|  | | *Individuals* | *Tracks* |
| *Wild Eastern* | | 46 | 111 |
| *Wild Central* | | 6 | 8 |
| *Wild Western* | | 3 | 6 |

**Table S2.** Corrected Akaike Information Criterion (AICc) model selection table for Generalised Linear Mixed Models (GLMM) with individual ID as a random effect testing the effect of population on **initial migratory orientation** of satellite tracked wild Asian houbara. Population is a three-level categorical variable denoting three populations within Uzbekistan (Eastern, Central, Western). Null is the intercept only model. Models ranked according to AICc and a *∆*AICc > 2 denotes a difference in models. Weight Top selected model is in bold. *W_i_* demotes model weight.

|  | *Model Selection* | | | | | | |
| --- | --- | --- | --- | --- | --- | --- | --- |
|  | | *Intercept* | *df* | *logLik* | *AICc* | *∆AICc* | *W_i_* |
| ***Null*** | | **203.50** | **3** | **-552.76** | **1111.70** | **0.00** | **0.88** |
| *Population* | | 203.60 | 5 | -552.65 | 1115.80 | 4.07 | 0.12 |

**Table S3**. Coefficient table from the **initial migratory orientation** GLMM including the categorical population variable with individual ID as a random effect.

| *Population Model Summary* | | | | | |
| --- | --- | --- | --- | --- | --- |
|  | *Estimate* | *Standard error* | *df* | *t* | *P* |
| *Intercept (Wild Eastern)* | 203.61 | 3.33 | 48.13 | 61.19 | <0.001 |
| Wild Central | 2.12 | 10.25 | 58.77 | 0.21 | 0.84 |
| Wild Western | -5.64 | 13.35 | 47.97 | -0.42 | 0.67 |

**Table S4**. Model estimated group differences from the **initial migratory orientation** GLMM including the categorical population variable with individual ID as a random effect.

| *Group Differences* | | | | |
| --- | --- | --- | --- | --- |
|  | *Estimate* | *Standard error* | *z* | *P* |
| *Wild Central vs Wild Eastern* | 2.12 | 10.25 | 0.21 | 0.98 |
| *Wild Western vs Wild Eastern* | -5.64 | 13.35 | -0.42 | 0.90 |
| *Wild Central vs Wild Western* | -7.76 | 16.16 | -0.48 | 0.88 |

**Table S5**. Pair-wise F-tests of variance in **initial migratory orientation** between three populations. P-values with Bonferroni correction for multiple comparisons are shown in the right hand column.

| *Variance tests* | | | | | |
| --- | --- | --- | --- | --- | --- |
|  | *F* | *Num df* | *Denom df* | *P* | *Bonferroni correction of P-value* |
| *Wild Central vs Wild Western* | 1.12 | 5 | 2 | 0.93 | 1 |
| *Wild Western vs Wild Eastern* | 0.79 | 45 | 2 | 0.58 | 1 |
| *Wild Central vs Wild Eastern* | 0.70 | 45 | 5 | 0.46 | 1 |

1. **Wintering site bearing**

**Table S6.** Sample sizes for **wintering site bearing** of satellite tracked wild Asian houbara from three populations within Uzbekistan showing the number of individuals monitored and the total number of complete migration tracks used in analysis.

|  | *Sample Sizes* | | |
| --- | --- | --- | --- |
|  | | *Individuals* | *Tracks* |
| *Wild Eastern* | | 50 | 124 |
| *Wild Central* | | 7 | 12 |
| *Wild Western* | | 3 | 6 |

**Table S7.** Corrected Akaike Information Criterion (AICc) model selection table for Generalised Linear Mixed Models (GLMM) with individual ID as a random effect testing the effect of population on **wintering site bearing** of satellite tracked wild Asian houbara. Population is a three-level categorical variable denoting three populations within Uzbekistan (Eastern, Central, Western). Null is the intercept only model. Models ranked according to AICc and a *∆*AICc > 2 denotes a difference in models. Weight Top selected model is in bold. *W_i_* demotes model weight.

|  | *Model Selection* | | | | | | |
| --- | --- | --- | --- | --- | --- | --- | --- |
|  | | *Int* | *df* | *logLik* | *AIC* | *∆AICc* | *W_i_* |
| ***Null*** | | **202.20** | **3** | **-514.79** | **1035.70** | **0.00** | **0.88** |
| *Population* | | 201.60 | 5 | -514.52 | 1039.80 | 3.74 | 0.13 |

**Table S8**. Coefficient table from the **wintering site bearing** including the categorical population variable with individual ID as a random effect.

| *Population Model Summary* | | | | | |
| --- | --- | --- | --- | --- | --- |
|  | *Estimate* | *Standard error* | *df* | *t* | *P* |
| *Intercept (Wild Eastern)* | 201.64 | 2.61 | 59.34 | 77.26 | <0.001 |
| Wild Central | 4.57 | 7.46 | 59.76 | 0.61 | 0.54 |
| Wild Western | -3.68 | 10.95 | 59.00 | -0.34 | 0.74 |

**Table S9**. Model estimated group differences from the **wintering site bearing** GLMM including the categorical population variable with individual ID as a random effect.

| *Group Differences* | | | | |
| --- | --- | --- | --- | --- |
|  | *Estimate* | *Standard error* | *z* | *P* |
| *Wild Central vs Wild Eastern* | 4.57 | 7.46 | 0.61 | 0.81 |
| *Wild Western vs Wild Eastern* | -3.68 | 10.95 | -0.34 | 0.94 |
| *Wild Central vs Wild Western* | -8.25 | 12.73 | -0.65 | 0.79 |

**Table S10**. Pair-wise F-tests of variance in **wintering site bearing** between three populations. P-values with Bonferroni correction for multiple comparisons are shown in the right hand column.

| *Variance tests* | | | | | |
| --- | --- | --- | --- | --- | --- |
|  | *F* | *Num df* | *Denom df* | *P* | *Bonferroni correction of P-value* |
| *Wild Central vs Wild Western* | 2.09 | 5 | 2 | 0.71 | 1 |
| *Wild Western vs Wild Eastern* | 2.44 | 45 | 2 | 0.67 | 1 |
| *Wild Central vs Wild Eastern* | 1.16 | 45 | 5 | 0.97 | 1 |

1. **Wintering site latitude**

**Table S11.** Sample sizes for **wintering site latitude** of satellite tracked wild Asian houbara from three populations within Uzbekistan showing the number of individuals monitored and the total number of complete migration tracks used in analysis.

|  | *Sample Sizes* | | |
| --- | --- | --- | --- |
|  | | *Individuals* | *Tracks* |
| *Wild Eastern* | | 50 | 125 |
| *Wild Central* | | 7 | 12 |
| *Wild Western* | | 3 | 6 |

**Table S12.** Corrected Akaike Information Criterion (AICc) model selection table for Generalised Linear Mixed Models (GLMM) with individual ID as a random effect testing the effect of population on **wintering site latitude** of satellite tracked wild Asian houbara. Population is a three-level categorical variable denoting three populations within Uzbekistan (Eastern, Central, Western). Null is the intercept only model. Models ranked according to AICc and a *∆*AICc > 2 denotes a difference in models. Weight Top selected model is in bold. *W_i_* demotes model weight.

|  | *Model Selection* | | | | | | |
| --- | --- | --- | --- | --- | --- | --- | --- |
|  | | *Intercept* | *df* | *logLik* | *AIC* | *∆AICc* | *W_i_* |
| ***Population*** | | **30.16** | **5** | **-293.65** | **597.70** | **0.00** | **1.00** |
| *Null* | | 31.07 | 3 | -307.06 | 620.30 | 22.57 | 0.00 |

**Table S13**. Coefficient table from the **wintering site latitude** GLMM including the categorical population variable with individual ID as a random effect.

| *Population Model Summary* | | | | | |
| --- | --- | --- | --- | --- | --- |
|  | *Estimate* | *Standard error* | *df* | *t* | *P* |
| *Intercept (Wild Eastern)* | 30.16 | 0.41 | 57.64 | 74.40 | <0.001 |
| Wild Central | 4.19 | 1.16 | 59.05 | 3.61 | <0.001 |
| Wild Western | 8.32 | 1.70 | 57.12 | 4.90 | <0.001 |

**Table S14**. Model estimated group differences from the **wintering site latitude** GLMM including the categorical population variable with individual ID as a random effect.

| *Group Differences* | | | | |
| --- | --- | --- | --- | --- |
|  | *Estimate* | *Standard error* | *z* | *P* |
| *Wild Central vs Wild Eastern* | 4.19 | 1.16 | 3.60 | <0.001 |
| *Wild Western vs Wild Eastern* | 8.32 | 1.70 | 4.90 | <0.001 |
| *Wild Central vs Wild Western* | 4.13 | 1.98 | 2.09 | 0.09 |

**Table S15**. Pair-wise F-tests of variance in **wintering site latitude** between three populations. P-values with Bonferroni correction for multiple comparisons are shown in the right hand column.

| *Variance tests* | | | | | |
| --- | --- | --- | --- | --- | --- |
|  | *F* | *Num df* | *Denom df* | *P* | *Bonferroni correction of P-value* |
| *Wild Central vs Wild Western* | 34.64 | 6 | 2 | 0.06 | 0.17 |
| *Wild Western vs Wild Eastern* | 11.80 | 49 | 2 | 0.16 | 0.49 |
| *Wild Central vs Wild Eastern* | 0.34 | 49 | 6 | 0.03 | 0.10 |

1. **Wintering site longitude**

**Table S16.** Sample sizes for **wintering site longitude** of satellite tracked wild Asian houbara from three populations within Uzbekistan showing the number of individuals monitored and the total number of complete migration tracks used in analysis.

| *Sample Sizes* | | |
| --- | --- | --- |
|  | *Individuals* | *Tracks* |
| *Wild Eastern* | 50 | 125 |
| *Wild Central* | 7 | 12 |
| *Wild Western* | 3 | 6 |

**Table S17.** Corrected Akaike Information Criterion (AICc) model selection table for Generalised Linear Mixed Models (GLMM) with individual ID as a random effect testing the effect of population on **wintering site longitude** of satellite tracked wild Asian houbara. Population is a three-level categorical variable denoting three populations within Uzbekistan (Eastern, Central, Western). Null is the intercept only model. Models ranked according to AICc and a *∆*AICc > 2 denotes a difference in models. Weight Top selected model is in bold. *W_i_* demotes model weight.

|  | *Model Selection* | | | | | | |
| --- | --- | --- | --- | --- | --- | --- | --- |
|  | | *Intercept* | *df* | *logLik* | *AIC* | *∆AICc* | *W_i_* |
| ***Population*** | | **59.87** | **5** | **-291.59** | **593.60** | **0.00** | **0.92** |
| *Null* | | 59.25 | 3 | -296.11 | 598.40 | 4.77 | 0.08 |
|  | |  |  |  |  |  |  |

**Table S18**. Coefficient table from the **wintering site longitude** GLMM including the categorical population variable with individual ID as a random effect.

| *Population Model Summary* | | | | | |
| --- | --- | --- | --- | --- | --- |
|  | *Estimate* | *Standard error* | *df* | *t* | *P* |
| *Intercept (Wild Eastern)* | 59.87 | 0.49 | 60.29 | 120.98 | <0.001 |
| Wild Central | -3.47 | 1.42 | 60.92 | -2.45 | 0.02 |
| Wild Western | -4.42 | 2.08 | 59.93 | -2.13 | 0.04 |

**Table S19**. Model estimated group differences from the **wintering site longitude** GLMM including the categorical population variable with individual ID as a random effect.

| *Group Differences* | | | | |
| --- | --- | --- | --- | --- |
|  | *Estimate* | *Standard error* | *z* | *P* |
| *Wild Central vs Wild Eastern* | -3.47 | 1.42 | -2.45 | 0.04 |
| *Wild Western vs Wild Eastern* | -4.42 | 2.08 | -2.13 | 0.08 |
| *Wild Central vs Wild Western* | -0.95 | 2.41 | -0.39 | 0.91 |

**Table S20**. Pair-wise F-tests of variance in **wintering site longitude** between three populations. P-values with Bonferroni correction for multiple comparisons are shown in the right hand column.

| *Variance tests* | | | | | |
| --- | --- | --- | --- | --- | --- |
|  | *F* | *Num df* | *Denom df* | *P* | *Bonferroni correction* |
| *Wild Central vs Wild Western* | 3.74 | 6 | 2 | 0.45 | 1.00 |
| *Wild Western vs Wild Eastern* | 3.52 | 49 | 2 | 0.49 | 1.00 |
| *Wild Central vs Wild Eastern* | 0.94 | 49 | 6 | 0.80 | 1.00 |

1. **Wintering site fidelity**

**Table S21.** Sample sizes for **wintering site fidelity** of satellite tracked wild Asian houbara from three populations within Uzbekistan showing the number of individuals monitored and the total number of complete migration tracks used in analysis.

| *Sample Sizes* | | |
| --- | --- | --- |
|  | *Individuals* | *Tracks* |
| *Wild Eastern* | 34 | 109 |
| *Wild Central* | 5 | 10 |
| *Wild Western* | 3 | 6 |

**Table S22.** Corrected Akaike Information Criterion (AICc) model selection table for Generalised Linear Mixed Models (GLMM) with individual ID as a random effect testing the effect of population on **wintering site fidelity** of satellite tracked wild Asian houbara. Population is a three-level categorical variable denoting three populations within Uzbekistan (Eastern, Central, Western). Null is the intercept only model. Models ranked according to AICc and a *∆*AICc > 2 denotes a difference in models. Weight Top selected model is in bold. *W_i_* demotes model weight.

|  | *Model Selection* | | | | | | |
| --- | --- | --- | --- | --- | --- | --- | --- |
|  | | *Intercept* | *df* | *logLik* | *AIC* | *∆AICc* | *W_i_* |
| ***Null*** | | **3.80** | **2** | **-75.61** | **155.50** | **0.00** | **0.87** |
| *Population* | | 3.72 | 4 | -75.16 | 159.40 | 3.87 | 0.13 |

**Table S23**. Coefficient table from the **wintering site fidelity** GLMM including the categorical population variable with individual ID as a random effect.

| *Population Model Summary (log transformed)* | | | | | |
| --- | --- | --- | --- | --- | --- |
|  | *Min* | *Max* | *Mean* | *SD* | *Variance* |
| *Intercept (Wild Eastern)* | 0.32 | 6.19 | 3.72 | 1.54 | 2.38 |
| Wild Central | 2.30 | 5.77 | 4.38 | 1.28 | 1.63 |
| Wild Western | 2.98 | 5.19 | 3.76 | 1.24 | 1.55 |

**Table S24**. Model estimated group differences from the **wintering site fidelity** GLMM including the categorical population variable with individual ID as a random effect.

| *Group Differences* | | | | |
| --- | --- | --- | --- | --- |
|  | *Estimate* | *Standard error* | *z* | *P* |
| *Wild Central vs Wild Eastern* | -3.47 | 1.42 | -2.45 | 0.04 |
| *Wild Western vs Wild Eastern* | -4.42 | 2.08 | -2.13 | 0.08 |
| *Wild Central vs Wild Western* | -0.95 | 2.41 | -0.39 | 0.91 |

**Table S25**. Pair-wise F-tests of variance in **wintering site fidelity** between three populations. P-values with Bonferroni correction for multiple comparisons are shown in the right hand column.

| *Variance tests* | | | | | |
| --- | --- | --- | --- | --- | --- |
|  | *F* | *Num df* | *Denom df* | *P* | *Bonferroni correction of P-value* |
| *Wild Central vs Wild Western* | 1.06 | 4 | 2 | 0.92 | 1.00 |
| *Wild Western vs Wild Eastern* | 1.54 | 33 | 2 | 0.94 | 1.00 |
| *Wild Central vs Wild Eastern* | 1.46 | 33 | 4 | 0.79 | 1.00 |

1. **Breeding site fidelity**

**Table S26.** Sample sizes for **breeding site fidelity** of satellite tracked wild Asian houbara from three populations within Uzbekistan showing the number of individuals monitored and the total number of complete migration tracks used in analysis.

| *Sample Sizes* | | |
| --- | --- | --- |
|  | *Individuals* | *Tracks* |
| *Wild Eastern* | 45 | 110 |
| *Wild Central* | 5 | 10 |
| *Wild Western* | 3 | 5 |

**Table S27.** Corrected Akaike Information Criterion (AICc) model selection table for Generalised Linear Mixed Models (GLMM) with individual ID as a random effect testing the effect of population on **breeding site fidelity** of satellite tracked wild Asian houbara. Population is a three-level categorical variable denoting three populations within Uzbekistan (Eastern, Central, Western). Null is the intercept only model. Models ranked according to AICc and a *∆*AICc > 2 denotes a difference in models. Weight Top selected model is in bold. *W_i_* demotes model weight.

|  | *Model Selection* | | | | | | |
| --- | --- | --- | --- | --- | --- | --- | --- |
|  | | *Intercept* | *df* | *logLik* | *AIC* | *∆AICc* | *W_i_* |
| ***Null*** | | **2.37** | **3** | **-224.77** | **455.70** | **0.00** | **0.80** |
| *Population* | | 2.46 | 5 | -224.00 | 458.50 | 2.76 | 0.20 |

**Table S28**. Coefficient table from the **breeding site fidelity** GLMM including the categorical population variable with individual ID as a random effect.

| *Population Model Summary* | | | | | |
| --- | --- | --- | --- | --- | --- |
|  | *Estimate* | *Standard error* | *df* | *t* | *P* |
| *Intercept (Wild Eastern)* | 2.46 | 0.20 | 51.76 | 12.31 | <0.001 |
| Wild Central | -0.31 | 0.63 | 56.19 | -0.49 | 0.62 |
| Wild Western | -0.99 | 0.84 | 64.33 | -1.19 | 0.24 |

**Table S29**. Model estimated group differences from the **breeding site fidelity** GLMM including the categorical population variable with individual ID as a random effect.

| *Group Differences* | | | | | | | |
| --- | --- | --- | --- | --- | --- | --- | --- |
|  | | | *Estimate* | *Standard error* | | *z* | *P* |
| *Wild Central vs Wild Eastern* | | | -0.31 | 0.63 | | -0.49 | 0.87 |
| *Wild Western vs Wild Eastern* | | | -0.99 | 0.84 | | -1.19 | 0.45 |
| *Wild Central vs Wild Western* | | | -0.68 | 1.01 | | -0.97 | 0.77 |
|  |  |  | | |  |  |  |

**Table S30**. Pair-wise F-tests of variance in **breeding site fidelity** between three populations. P-values with Bonferroni correction for multiple comparisons are shown in the right hand column.

| *Variance tests* | | | | | |
| --- | --- | --- | --- | --- | --- |
|  | *F* | *Num df* | *Denom df* | *P* | *Bonferroni correction of P-value* |
| *Wild Central vs Wild Western* | 0.54 | 4 | 2 | 0.54 | 1.00 |
| *Wild Western vs Wild Eastern* | 1.96 | 44 | 2 | 0.79 | 1.00 |
| *Wild Central vs Wild Eastern* | 3.63 | 44 | 4 | 0.22 | 0.65 |

1. **Straight line distance**

**Table S31.** Sample sizes for **straight line distance** of satellite tracked wild Asian houbara from three populations within Uzbekistan showing the number of individuals monitored and the total number of complete migration tracks used in analysis.

| *Sample Sizes* | | |
| --- | --- | --- |
|  | *Individuals* | *Tracks* |
| *Wild Eastern* | 50 | 124 |
| *Wild Central* | 7 | 12 |
| *Wild Western* | 3 | 6 |

**Table S32.** Corrected Akaike Information Criterion (AICc) model selection table for Generalised Linear Mixed Models (GLMM) with individual ID as a random effect testing the effect of population on **straight line distance** of satellite tracked wild Asian houbara. Population is a three-level categorical variable denoting three populations within Uzbekistan (Eastern, Central, Western). Null is the intercept only model. Models ranked according to AICc and a *∆*AICc > 2 denotes a difference in models. Weight Top selected model is in bold. *W_i_* demotes model weight.

|  | *Model Selection* | | | | | | |
| --- | --- | --- | --- | --- | --- | --- | --- |
|  | | *Intercept* | *df* | *logLik* | *AIC* | *∆AICc* | *W_i_* |
| ***Population*** | | **1184** | **5** | **-991.96** | **1994.35** | **0.00** | **0.90** |
| *Null* | | 1140 | 3 | -996.24 | 1998.66 | 4.31 | 0.10 |

**Table S33**. Coefficient table from the **straight line distance** GLMM including the categorical population variable with individual ID as a random effect.

| *Population Model Summary* | | | | | |
| --- | --- | --- | --- | --- | --- |
|  | *Estimate* | *Standard error* | *df* | *t* | *P* |
| *Intercept (Wild Eastern)* | 1183.68 | 43.81 | 59.67 | 27.02 | <0.001 |
| Wild Central | -153.93 | 126.85 | 63.40 | -1.22 | 0.23 |
| Wild Western | -527.52 | 183.08 | 59.43 | -2.88 | <0.01 |

**Table S34**. Model estimated group differences from the **straight line distance** GLMM including the categorical population variable with individual ID as a random effect.

| *Group Differences* | | | | | | | |
| --- | --- | --- | --- | --- | --- | --- | --- |
|  | | | *Estimate* | *Standard error* | | *z* | *P* |
| *Wild Central vs Wild Eastern* | | | -153.93 | 126.65 | | -1.22 | 0.43 |
| *Wild Western vs Wild Eastern* | | | -527.52 | 183.08 | | -2.88 | 0.01 |
| *Wild Central vs Wild Western* | | | -373.58 | 213.83 | | -1.75 | 0.18 |
|  |  |  | | |  |  |  |

**Table S35**. Pair-wise F-tests of variance in **straight line distance** between three populations. P-values with Bonferroni correction for multiple comparisons are shown in the right hand column.

| *Variance tests* | | | | | |
| --- | --- | --- | --- | --- | --- |
|  | *F* | *Num df* | *Denom df* | *P* | *Bonferroni correction of P-value* |
| *Wild Central vs Wild Western* | 32.61 | 6 | 2 | 0.06 | 0.18 |
| *Wild Western vs Wild Eastern* | 5.12 | 49 | 2 | 0.35 | 1.00 |
| *Wild Central vs Wild Eastern* | 0.16 | 49 | 6 | <0.001 | <0.001 |

1. **Departure date**

**Table S36.** Sample sizes for **departure date** of satellite tracked wild Asian houbara from three populations within Uzbekistan showing the number of individuals monitored and the total number of complete migration tracks used in analysis.

| *Sample Sizes* | | |
| --- | --- | --- |
|  | *Individuals* | *Tracks* |
| *Wild Eastern* | 32 | 47 |
| *Wild Central* | 6 | 8 |
| *Wild Western* | 3 | 6 |

**Table S37.** Corrected Akaike Information Criterion (AICc) model selection table for Generalised Linear Mixed Models (GLMM) with individual ID as a random effect testing the effect of population on **departure date** of satellite tracked wild Asian houbara. Population is a three-level categorical variable denoting three populations within Uzbekistan (Eastern, Central, Western). Null is the intercept only model. Models ranked according to AICc and a *∆*AICc > 2 denotes a difference in models. Weight Top selected model is in bold. *W_i_* demotes model weight.

|  | *Model Selection* | | | | | | |
| --- | --- | --- | --- | --- | --- | --- | --- |
|  | | *Intercept* | *df* | *logLik* | *AIC* | *∆AICc* | *W_i_* |
| ***Null*** | | **290.30** | **3** | **-275.46** | **577.34** | **0.00** | **0.59** |
| *Population* | | 288.00 | 5 | -273.47 | 558.03 | 0.69 | 0.41 |

**Table S38**. Coefficient table from the **departure date** GLMM including the categorical population variable with individual ID as a random effect.

| *Population Model Summary* | | | | | |
| --- | --- | --- | --- | --- | --- |
|  | *Estimate* | *Standard error* | *df* | *t* | *P* |
| *Intercept (Wild Eastern)* | 288.00 | 3.83 | 38.42 | 75.14 | <0.001 |
| Wild Central | 18.92 | 9.78 | 40.76 | 1.94 | 0.06 |
| Wild Western | -4.84 | 12.42 | 30.77 | -0.39 | 0.70 |

**Table S39**. Model estimated group differences from the **departure date** GLMM including the categorical population variable with individual ID as a random effect.

| *Group Differences* | | | | | | | |
| --- | --- | --- | --- | --- | --- | --- | --- |
|  | | | *Estimate* | *Standard error* | | *z* | *P* |
| *Wild Central vs Wild Eastern* | | | 18.92 | 9.78 | | 1.94 | 0.12 |
| *Wild Western vs Wild Eastern* | | | -4.84 | 12.42 | | -0.39 | 0.92 |
| *Wild Central vs Wild Western* | | | -23.76 | 14.85 | | -1.60 | 0.24 |
|  |  |  | | |  |  |  |

**Table S40**. Pair-wise F-tests of variance in **departure date** between three populations. P-values with Bonferroni correction for multiple comparisons are shown in the right hand column.

| *Variance tests* | | | | | |
| --- | --- | --- | --- | --- | --- |
|  | *F* | *Num df* | *Denom df* | *P* | *Bonferroni correction of P-value* |
| *Wild Central vs Wild Western* | 7.46 | 5 | 2 | 0.24 | 0.73 |
| *Wild Western vs Wild Eastern* | 13.07 | 31 | 2 | 0.15 | 0.44 |
| *Wild Central vs Wild Eastern* | 1.75 | 31 | 5 | 0.56 | 1.00 |
