# Supplementary material for: Releases of Asian houbara must respect genetic and geographic origin to preserve inherited migration behaviour: evidence from a translocation experiment
Source: R Soc Open Sci. 2020 Mar 18;7(3):200250. doi: 10.1098/rsos.200250 (PMC7137974; doi:10.1098/rsos.200250)
Supplement: Supplementary methods [file rsos200250supp2.docx]

**Supplementary Material**

**Inherited behaviour of translocated animals risks altering migration patterns in recipient populations.**

**Methods:**

**Details of Asian houbara capture, satellite telemetry and telemetry data processing**

**Capture of wild Houbara**

Houbara were captured using leg-snares following Seddon et al. (1999) with males (at least two years old) caught on established display sites and females (at least one year old) caught at the nest, so that all wild individuals tracked would previously have completed at least one return migration. Between autumn 2011 and spring 2019, 50 wild adults (female = 37, male = 13) were tagged in the eastern population, while between 16 March‒17 May 2017, only males were trapped in the central (n = 7) and western (n = 3) populations as it was not possible to locate females in these areas that lack the extensive sandy substrates required for nest finding by following female tracks (Guilherme et al. 2018).

**Egg collection and headstarting**

In spring 2017, eleven wild clutches were collected from the eastern population and transported (using portable incubators) to the Emirates Bird Breeding Centre for Conservation (EBBCC) where they were artificially incubated and hand-reared in captivity. Young birds were raised in the absence of adult houbara (in contrast to wild juveniles that pass two months with maternal supervision) in groups of six per cage (larger than a natural brood size of 2-4). Birds were reared on wheat pellets designed to meet their nutrition growth needs with diet later supplemented with leafy vegetation and live food. As birds grew they progressed into larger housing, but lacked any opportunity to practice flying, as the risk of injury during this activity was too great. Previous research tracking released captive-bred juveniles and yearlings in the eastern population confirmed that rearing protocols support sufficient physiological development for released birds to successfully migrate (Burnside et al. 2017). However, although captive-bred and captive-reared individuals showed similar migratory orientation as wild juveniles and adults, and similar stop duration and migratory efficiency (deviation of the straightest path) to wild juveniles, they migrated 20.6 days (4.6 SE) later and consequently settled in wintering sites located 470.8 km (76.4se) further north than wild juveniles. So if captive-rearing affects subsequent migratory behaviour, we may expect translocated birds to travel a lesser distance than wild adults. Translocation releases occurred on 19 and 21 September 2017 into central and western populations, respectively.

Birds were transported in crates to the release sites that were areas used by wild adults tracked in the western and central populations. Translocated birds were released with no subsequent support (‘hard release’).

**Satellite telemetry & data processing**

All monitored birds were fitted with backpack harness mounted satellite transmitters (Microwave Telemetry PTT either 30g or 45g Argos/GPS PTT-100 models). For each animal track, the distance, interval duration and speed (distance/interval) travelled between sequential GPS fixes (steps) was calculated using adehabitatLT (Callenge 2006) in R software (R Core Team 2019) and step behaviours were classified using speed as: foraging (< 2 kph) or transit (> 2 kph), following Burnside et al. (2017).

Asian Houbara migration is characterised by a series of ‘migration movements’ linked by ‘stopovers’ that last a mean of 2.1 days ± 0.3 SE, before commencing the next migration movement on a similar trajectory to the previous migration movement (Burnside et al. 2017). Migration movements are displacements much larger (mean distance between stops = 228 ± 22 km, Burnside et al. 2017) than those seen during the post-breeding homerange in adults or the natal dispersal of juveniles. For example, houbara have a post-breeding period (after finishing displaying, nesting or leaving chicks) starting on the 20^th^ June ± 16.2 days SD for males and 5^th^ July ± 24.3 days SD for females during which they undergo a lengthy moulting period (up to 20 weeks to complete, Saint-Jalme et al. 1996). Their post-breeding home range is on average 2617-3215 km^2^ (equivalent to a *c* 54*54 km square) until migration (Koshkin et al. 2016). Juvenile movements tend to be small with a net dispersal distance of 9.1-16.2km in the three months after fledging (June-July) from their natal area (Hardouin et al. 2012).

To estimate initial orientation during the first migration step, it was necessary to identify the first migration movement from the post-breeding area to arrival at the first stopover. To delineate the start of migration (last fix on the summering area), behavioural transitions from foraging to transit after August were defined as onset of migration if accompanied by a step comparable to a migration movement away from the post-breeding/release area again followed by a series of stopovers and migration movements on a similar trajectory. To reliably identify stopover behaviour required at least three fixes per day to show consecutive foraging movements; the median number of fixes per day was 4.8 (IQR: 4.24‒5.08) and tracks with fewer than three fixes per day were excluded from estimates of initial migratory orientation.

All complete tracks provided a wintering-site bearing, estimated as the angle between the last fix on the post-breeding site and the first fix on the wintering-site. Identifying the wintering-site was straightforward for birds that survived to make a return migration, with the arrival point defined as the last behavioural transition from transit to foraging at the wintering site farthest from the post-breeding area. For birds that did not survive the winter we judged whether they had reached their wintering-site before dying, or were potentially only at a stopover, using a threshold of > 14 continuous days (the upper 95-tile of stopover duration) of foraging movements; tracks of individuals that had been foraging without migratory movement for fewer days before dying were classed as providing incomplete migration (Burnside et al. 2017) and were therefore excluded from estimates of wintering-site bearing, wintering-site longitude and latitude, but provided initial bearing. For individuals that survived more than one migration, wintering-site fidelity was quantified as an individuals’ mean pairwise straight-line distance (km) between all (not sequential) wintering-sites. Fidelity to breeding/release site was quantified in terms of straight-line distance (km) between the adult (breeding-ground) capture location or captive-reared translocated release location and the end of the return migration track.

**References**

Burnside R J, Collar NJ, Dolman PM. 2017. Comparative migration strategies of wild and captive-bred Asian houbara *Chlamydotis macqueenii*. Ibis **159**: 374-389.

Calenge, C. 2006. The package Adehabitat for the R software: a tool for the analysis of space and habitat use by animals. Ecological modelling **197**: 516-519.

Guilherme JL, Burnside RJ, Collar NJ, Dolman PM. 2018. Consistent nest-site selection across habitats increases fitness in Asian Houbara. Auk **135**:192-205.

Hardouin LA, Nevoux M, Robert A, Gimenez O, Lacroix F, Hingrat Y. 2012. Determinants and costs of natal dispersal in a lekking species. Oikos **121**:804-812.

Koshkin M. 2016. Habitat, abundance and productivity of the Asian Houbara *Chlamydotis* *macqueenii* in Uzbekistan. PhD Thesis, University of East Anglia, Norfolk, UK.

R Core Team. 2013. R: A language and environment for statistical computing. R Foundation for Statistical Computing, Vienna, Austria.

Saint Jalme M, Williams J, Mikaelian I, Gaucher P, Paillat P. 1996. Seasonal variation of LH, sex steroids, body mass, moult, display and laying in two subspecies of houbara bustard *Chlamydotis undulata undulata* and *Chlamydotis undulata macqueenii* housed in outdoor cages under natural conditions. General and Comparative Endocrinology **102**:102-112.
