# Supplementary material for: Releases of Asian houbara must respect genetic and geographic origin to preserve inherited migration behaviour: evidence from a translocation experiment
Source: R Soc Open Sci. 2020 Mar 18;7(3):200250. doi: 10.1098/rsos.200250 (PMC7137974; doi:10.1098/rsos.200250)
Supplement: Supplementary Figure [file rsos200250supp3.docx]

**Supplementary Material**

**Inherited behaviour of translocated animals risks altering migration patterns in recipient populations.**

**Figure S3:**

**
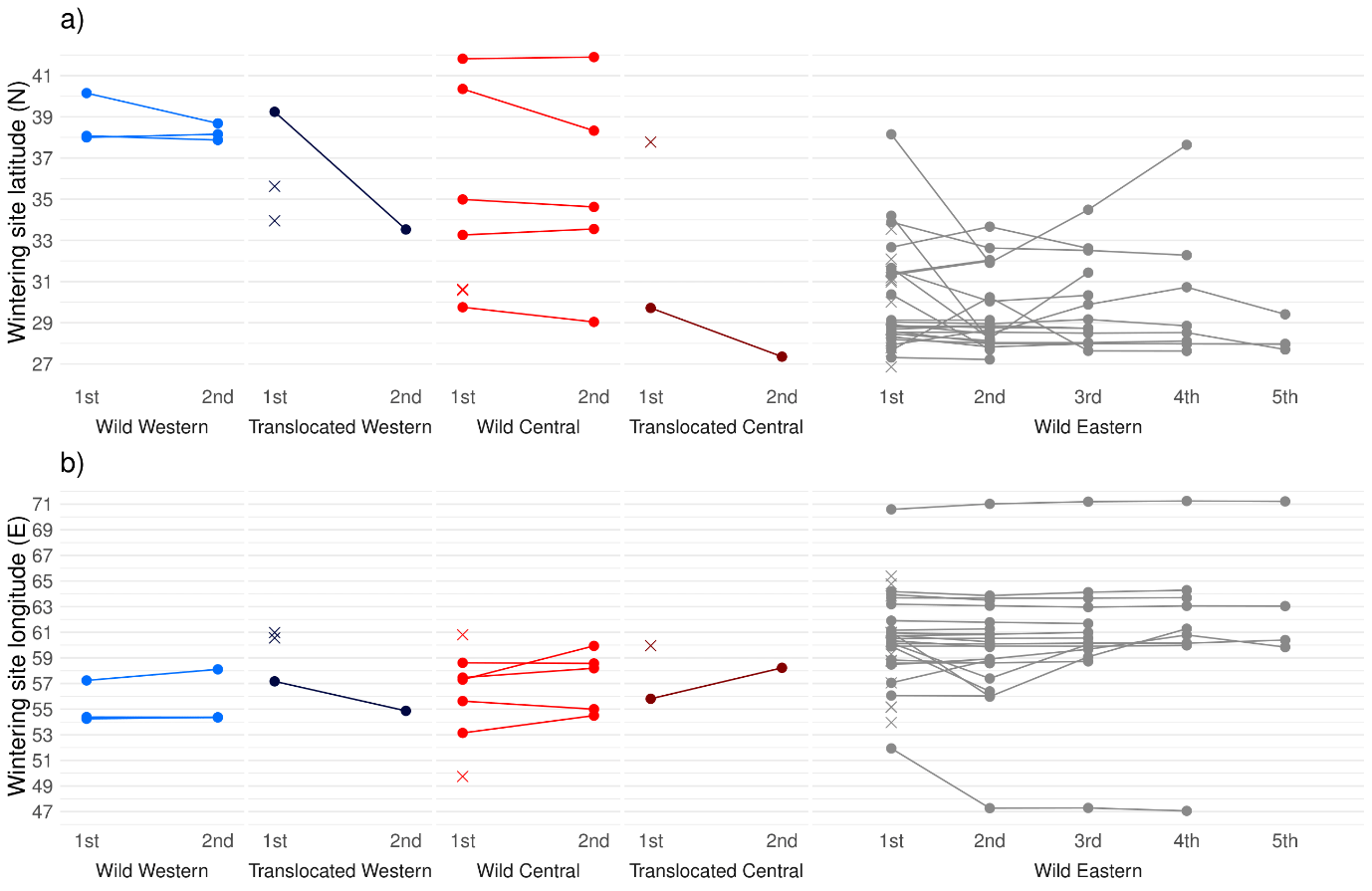
**

**Figure S1.** Inter-individual consistency in successive wintering-site latitudes (a) and longitudes (b) of wild and translocated Asian Houbara. Shown are wild adult individuals sampled from 3 populations (Western, Central, and Eastern Uzbekistan) and 2 groups translocated from the eastern source into the central and western population ranges. Individuals that only had a record of one wintering site are denoted with an x.
