# Supplementary material for: Releases of Asian houbara must respect genetic and geographic origin to preserve inherited migration behaviour: evidence from a translocation experiment
Source: R Soc Open Sci. 2020 Mar 18;7(3):200250. doi: 10.1098/rsos.200250 (PMC7137974; doi:10.1098/rsos.200250)
Supplement: Reviewer comments [file rsos200250_review_history.pdf]

THE ROYAL SOCIETY
PUBLISHING

ROYAL SOCIETY
OPEN SCIENCE

Releases of Asian houbara must respect genetic and geographic origin to preserve inherited migration behaviour: evidence from a translocation experiment

Robert J. Burnside, Claire Buchan, Daniel Salliss, Nigel J. Collar and Paul M. Dolman

Article citation details
R. Soc. open sci. **7**: 200250.
http://dx.doi.org/10.1098/rsos.200250

Review timeline
Original submission: 18 February 2020
Final acceptance: 26 February 2020

Note: Reports are unedited and appear as submitted by the referee. The review history appears in chronological order.

Note: This manuscript was transferred from another Royal Society journal with peer review.

Review History

Decision letter (RSOS-200250.R0)

26-Feb-2020

Dear Dr Burnside:

It is a pleasure to accept your manuscript entitled "Releases of Asian Houbara must respect genetic & geographic origin to preserve inherited migration behaviour: evidence from a translocation experiment" in its current form for publication in Royal Society Open Science.

Please ensure that you send to the editorial office an editable version of your accepted manuscript, and individual files for each figure and table included in your manuscript. You can send these in a zip folder if more convenient. Failure to provide these files may delay the processing of your proof. You may disregard this request if you have already provided these files to the editorial office.

You can expect to receive a proof of your article in the near future. Please contact the editorial office (openscience_proofs@royalsociety.org) and the production office (openscience@royalsociety.org) to let us know if you are likely to be away from e-mail contact -- if you are going to be away, please nominate a co-author (if available) to manage the proofing process, and ensure they are copied into your email to the journal.

Reports © 2020 The Reviewers; Decision Letters © 2020 The Reviewers and Editors; Responses © 2020 The Reviewers, Editors and Authors. Published by the Royal Society under the terms of the Creative Commons Attribution License http://creativecommons.org/licenses/by/4.0/, which permits unrestricted use, provided the original author and source are credited

Due to rapid publication and an extremely tight schedule, if comments are not received, your paper may experience a delay in publication.

Please see the Royal Society Publishing guidance on how you may share your accepted author manuscript at https://royalsociety.org/journals/ethics-policies/media-embargo/.

Thank you for your fine contribution. On behalf of the Editors of Royal Society Open Science, we look forward to your continued contributions to the Journal.

Kind regards,
Anita Kristiansen
Editorial Coordinator

Royal Society Open Science
openscience@royalsociety.org

on behalf of Dr Oliver Schülke (Associate Editor) and Professor Pete Smith (Subject Editor).

Associate Editor Dr Oliver Schülke Comments to Author:
Comments to the Author:
The authors did a great job in revising the ms in accordance with the reviewers' comments and made it very easy for me as a new handling editor to evaluate their work.

Follow Royal Society Publishing on Twitter: @RSocPublishing
Follow Royal Society Publishing on Facebook:
https://www.facebook.com/RoyalSocietyPublishing.FanPage/
Read Royal Society Publishing's blog: https://blogs.royalsociety.org/publishing/
